# Enhancing the Figure of Merit of Heavy‐Band Thermoelectric Materials Through Hierarchical Phonon Scattering

**DOI:** 10.1002/advs.201600035

**Published:** 2016-03-15

**Authors:** Chenguang Fu, Haijun Wu, Yintu Liu, Jiaqing He, Xinbing Zhao, Tiejun Zhu

**Affiliations:** ^1^ State Key Laboratory of Silicon MaterialsSchool of Materials Science and EngineeringZhejiang UniversityHangzhou310027P.R. China; ^2^ Department of PhysicsSouth University of Science and Technology of ChinaShenzhen518055P.R. China; ^3^ Department of Materials Science and EngineeringNational University of Singapore7 Engineering Drive 1Singapore117575Singapore

**Keywords:** half‐Heusler alloys, semiconductors, thermoelectrics

## Abstract

**Hierarchical scattering** is suggested as an effective strategy to enhance the figure of merit *zT* of heavy‐band thermoelectric materials. Heavy‐band FeNbSb half‐Heusler system with intrinsically low carrier mean free path is demonstrated as a paradigm. An enhanced *zT* of 1.34 is obtained at 1150 K for the Fe_1.05_Nb_0.75_Ti_0.25_Sb compound with intentionally designed hierarchical scattering centers.

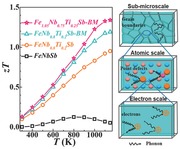

In the past decades, thermoelectric (TE) materials have received rejuvenated interest due to their promising application in direct thermal to electric energy conversion and solid‐refrigeration that could lead to significant energy savings,^1^, ^2^ and also in other technological fields such as sensors and thermopower wave sources.^3^, ^4^ The efficiency of a TE material is gauged by the dimensionless figure of merit, *zT* = *α* ^2^ *σT*/(*κ*_e_+*κ*_L_), where *α*, *σ*, *T*, *κ*_e_ and *κ*_L_ are, respectively, the Seebeck coefficient, the electrical conductivity, the absolute temperature, and the electronic and lattice components of total thermal conductivity *κ*.^5^ Large‐scale application of TE technology demands the materials having high *zT* values, which however are difficult to achieve due to the interdependencies of *α*, *σ*, and *κ_e_* via the carrier concentration.^5^ Besides, the decrease in *κ* by introducing more phonon scattering centers may concomitantly scatter the carriers leading to a decrease in *σ*.^6^ Therefore, strategies which can decouple these parameters for synergistic optimization of electron and phonon transport are highly desirable and central theme for high *zT*.

Current efforts to improve *zT* are focused on the optimization of power factor *α* ^2^ *σ* through controlled doping or electron band engineering,^7^, ^8^, ^9^, ^10^, ^11^ suppression in *κ*_L_ by alloying or nanostructuring,^1^, ^2^, ^5^, ^6^ and development of new materials with intrinsically low *κ*_L_.^12^, ^13^, ^14^, ^15^ Heat‐carrying phonons cover a broad spectrum of frequency, and the *κ*_L_ is a sum of contributions from phonons of different frequencies. Thus, introducing hierarchical phonon scattering centers into the matrix, which could lead to a substantially reduced *κ*_L_, has recently been proposed to improve the TE performance of traditional PbTe‐ and B_2_Te_3_‐based materials.^1^, ^16^, ^17^ However, the charge transport may also be significantly diminished by the introduced scattering centers, offsetting the beneficial contribution to *zT*.^18^ Therefore, maintaining high carrier mobility *μ* while greatly suppressing *κ*_L_ is a key to improve the TE properties by introducing hierarchical scattering centers. To make this approach effective, the hierarchical scattering centers should be smaller than the phonon mean free path (*l*_ph_) but larger than the carrier mean free path (*l*_c_), so that phonons are more strongly scattered than carriers.^6^ Therefore, the *zT* of TE materials with intrinsically high *l*_ph_ and low *l*_c_ are more likely to be improved by using this approach. This can be most possibly realized in heavy‐band TE materials with large band effective mass and low charge mobility, such as GeTe‐based materials and p‐type (Hf,Zr)CoSb‐based half‐Heusler (HH) compounds.^19^, ^20^, ^21^

Among various kinds of state‐of‐the‐art TE materials, HH compounds distinguish themselves by excellent power factors but intrinsically high *κ*_L_,^18^, ^22^ which respectively result from the sharp density of states near the Fermi level and the simple crystal structure.^23^, ^24^ To reduce the *κ*_L_ of HH compounds, isoelectronic alloying and nanostructuring are widely used,^25^, ^26^, ^27^, ^28^, ^29^, ^30^ which however may also cause a deterioration in *μ*, offsetting the reduction in *κ*_L_ to some extent. For example, the average *l*_c_ and *l*_ph_ for n‐type Ni*M*Sn (*M* = Hf, Zr, Ti) HH system are of the same order (≈10^−9^ m) above room temperature, and the *zT* enhancement based on grain refinement is hence quite limited.^18^ Similar result is also found for n‐type FeVSb‐based HH alloys.^27^ For these n‐type HH compounds the single band effective mass *m*_b_* are usually in the range of 1.0–1.3 *m*_e_.^27^, ^28^ In contrast, high *m*_b_* of ≈2.5 *m*_e_ are found for the p‐type Fe(V,Nb)Sb and ZrCoSb‐based HH compounds.^29^, ^30^ The room temperature *μ* of these p‐type HH compounds is usually several times lower than that of the typical n‐type counterparts,^27^, ^28^, ^29^, ^30^ which usually means smaller *l*_c_ at the optimal carrier concentration. Hence, introducing more phonon scattering centers into the p‐type HH materials may be more feasible to improve their *zT*s, compared with the n‐type counterparts.

Recently, p‐type FeNbSb‐based HH compounds have been identified as promising TE materials with high *zT* > 1 at high temperatures.^31^, ^32^ Through high content of Ti and Hf dopants, the maximum *zT* of ≈1.1 at 1100 K and ≈1.5 at 1200 K can be obtained respectively for FeNb_0.8_Ti_0.2_Sb and FeNb_0.86_Hf_0.14_Sb. For large‐scale applications, low materials cost is as important as high *zT*. Compared with the high price of Hf (≈579 USD kg^−1^), the cost of Ti (≈11.6 USD kg^−1^) is much lower.^26^, ^33^ Ti is also one of the most abundant elements in the earth's crust (6600 p.p.m.), about three orders of magnitude higher than Hf (3.3 p.p.m.).^34^ Hence, from the view of materials cost and element abundance, Ti‐doped FeNbSb system is more attractive for practical application, and further improving the *zT* of Ti‐doped FeNbSb will greatly enhance the feasibility. Heavily doped p‐type FeNb_1−_ *_x_*Ti*_x_*Sb with high *m*_b_* have relatively low *μ* of ≈15 cm^2^ V^−1^ s^−1^ at 300 K,^31^ implying the existence of low *l_c_*. The experimentally obtained lowest *κ*_L_ for p‐type FeNb_1−_ *_x_*Ti*_x_*Sb in the previous work is ≈2.6 Wm^−1^ K^−1^ at 1100 K,^31^ higher than the calculated theoretical minimum value (≈1 Wm^−1^ K^−1^), indicating that further *κ*_L_ suppression would be one of the main directions to improve the *zT*. Introducing hierarchical scattering centers into this p‐type HH system may be favorable for higher *zT*.

In this work, it is found that the *l*_c_ in the p‐type FeNbSb is comparable to the lattice parameter, indicating that the carrier mobility of this system almost reaches the Ioffe–Regel limit,^35^ which means that the carrier scattering has reached the highest limit and introducing more phonon scattering centers will not impair the power factor while largely suppress the *κ*_L_. By synergistically doping high content of Ti, refining the grain size to sub‐microscale, and introducing more point defects into the matrix, the *κ*_L_ is largely decreased while the power factor is only slightly affected. As a result, an enhanced *zT* of 1.34 is obtained at 1150 K for the intentionally designed Fe_1.05_Nb_0.75_Ti_0.25_Sb HH compound. These results highlight the efficacy of hierarchical phonon scattering in improving the performance of heavy‐band TE materials.

For the p‐type FeNbSb, 20% Ti‐doped FeNb_0.8_Ti_0.2_Sb has the optimal carrier concentration.^31^ High content of Ti dopant not only supplies enough carriers for the optimal power factor but also creates strong mass and strain field fluctuations and electron‐phonon scattering leading to great suppression in *κ*_L_. How to further reduce the *κ*_L_ in FeNb_0.8_Ti_0.2_Sb? As schematically shown in **Figure** **1**a, the answer is to introduce hierarchical phonon scattering centers to scatter the phonons with different frequencies. The sub‐microscale grain boundaries, usually targeting the low frequency phonons, can be introduced into the matrix via the ball‐milling process.^6^, ^36^ In addition, more atomic‐scale point defects can be created to further enhance the scattering of high‐frequency phonons. Thus, by combining the submicroscale grain boundaries, atomic‐scale point defects and electron–phonon interaction, hierarchical phonon scattering centers can be concurrently created in a given material, leading to a substantially reduced *κ*_L_.^1^, ^37^, ^38^

**Figure 1:**
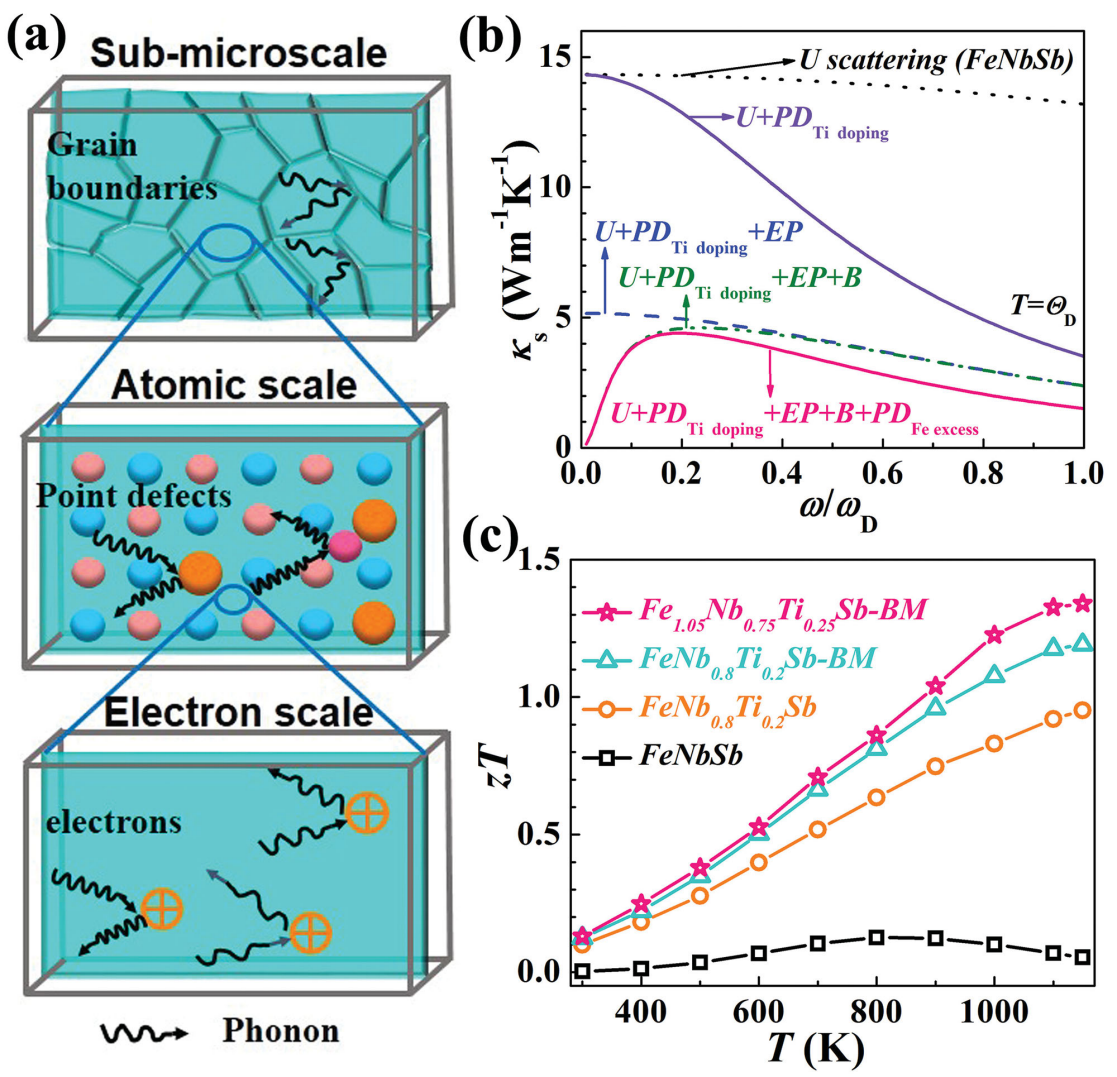
a) A schematic illustration showing hierarchical phonon scattering. The low frequency (LF), high frequency (HF) and full frequency (FF) phonons are respectively scattered by the sub‐microscale grain boundaries (B), point defects (PD) and electrons (EP). b) Phonon frequency dependence of spectral lattice thermal conductivity *κ*_s_ for the samples with different phonon scattering mechanisms (see Supporting Information). U represents phonon–phonon Umklapp process and *ω*_D_ is Debye frequency. PD_Ti doping_ and PD_Fe excess_ donate the point defect scattering due to Ti doping and Fe excess, respectively. c) Temperature dependence of *zT* for p‐type FeNbSb system with intentionally designed hierarchical phonon scattering centers.

Figure 1b shows the calculated spectral lattice thermal conductivity for the so‐made Ti‐doped FeNbSb compounds with hierarchical phonon scattering centers (see details in Supporting Information). High content of Ti dopant in FeNbSb induces strong mass/strain field fluctuation and electron–phonon interaction, which contribute to a broad frequency scattering of phonons and result in a ≈70% reduction compared to the *κ*_L_ of FeNbSb. Furthermore, the introduced grain boundaries by ball milling scatter long‐wavelength phonons and reduce *κ*_L_. Excess Fe, which may enter into the tetrahedral interstitial sites as excess Ni in ZrNiSn HH alloys,^39^ is added into the matrix to enhance the point defect scattering of phonons. With the introduced point defects, the *κ*_L_ is further suppressed resulting from the enhanced scattering of phonons with high frequencies as seen in Figure 1b.

All in all, by introducing the sub‐microscale grain boundaries, atomic‐scale point defects and electron–phonon interaction as hierarchical phonon scattering centers, a great reduction of ≈80% in the *κ*_L_ of FeNbSb has been achieved. Coupled with the unchanged electrical properties, a significantly enhanced peak *zT* of 1.34 have been experimentally obtained at 1150 K for the grain‐refined Fe_1.05_Nb_0.75_Ti_0.25_Sb, as presented in Figure 1c, which justifies the concept that introducing hierarchical phonon scattering centers into heavy‐band HH system is indeed an effective strategy to improve the *zT*.

The scanning electron microscopy (SEM) and transmission electron microscopy (TEM) analyses are performed to identify the microstructure features. The SEM images of the FeNb_0.8_Ti_0.2_Sb samples with different ball‐milling (BM) time are displayed in **Figure** **2**. The average particle size of the four samples milled for 1, 4, 8, and 16 h is estimated to be about 1.6 μm, 0.8 μm, 300 nm and 200 nm, respectively. It is worth noting that, although the BM time for the sample BM‐16h is largely prolonged, compared with that of sample BM‐8h, the decrease in average particle size is not as obvious as that of other samples with the shorter BM times. The TEM observation of the sample BM‐16h shows that the average grain size is about 140 nm (Figure 2e,f).

**Figure 2:**
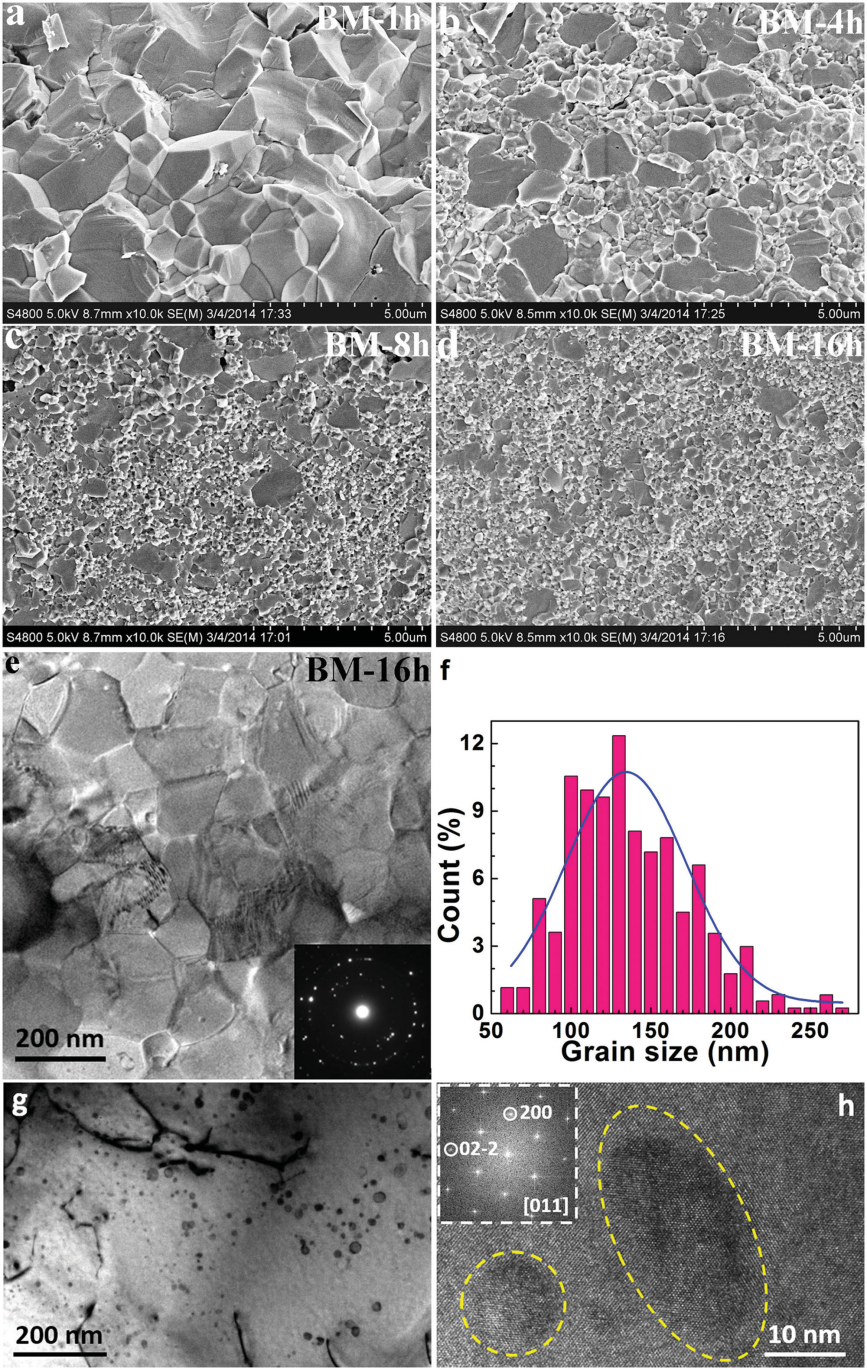
a–d) SEM images of the fractured surfaces for the FeNb_0.8_Ti_0.2_Sb samples with different BM time. e) Low‐magnification TEM image and f) grain size distribution histogram for the FeNb_0.8_Ti_0.2_Sb sample BM‐16h. The inset in e) shows the polycrystalline electron diffraction pattern. g) Low‐magnification TEM image showing the nanoscale precipitates and dark lines from defects in a typical region with relatively high density of precipitates and h) HRTEM image with an inserted FFT pattern for the nanoscale precipitates in sample Fe_1.05_Nb_0.75_Ti_0.25_Sb.

Figure 2g shows the low‐magnification TEM image for sample Fe_1.05_Nb_0.75_Ti_0.25_Sb. The spherically shaped and unevenly distributed nanoscale precipitates can be occasionally found in the matrix. Figure 2h gives the high‐resolution TEM (HRTEM) image with an inserted fast Fourier transferred (FFT) pattern for the nanoscale precipitates. The high‐angle annular dark field (HAADF) STEM in combination with energy‐dispersive X‐ray spectroscopy (EDS) was performed to identify the composition of the nanoscale precipitates (Figure S1, Supporting Information). The analysis shows that the nanoscale precipitates are Ti‐rich, resulting from the high Ti content beyond the solubility of Ti (≈23%) in FeNbSb (see details in Supporting Information). The Ti‐rich nanoscale precipitates are initially designed to enhance the scattering of middle frequency phonons. However, the results below will show they have weak effect on the electrical and thermal properties, which may result from the relatively small contents of precipitates in the matrix.

An important aspect making the hierarchical design effective in improving *zT* of a TE system, is the charge carrier transport cannot be significantly degraded. **Figure** **3**a shows the temperature dependence of electrical conductivity *σ* for the FeNb_0.8_Ti_0.2_Sb and Fe_1+_ *_x_*Nb_0.75_Ti_0.25_Sb samples. The *σ* of FeNb_0.8_Ti_0.2_Sb has only a slight decrease with increasing BM times, due to the trivial reduction in carrier concentration and carrier mobility (S1, Supporting Information). The carrier mean free path *l*_c_ of the FeNb_0.8_Ti_0.2_Sb samples are estimated by the formula *l*_c_ = (2*E*_F_ *m*_b_*)^1/2^ *μ*/*e*, where the Fermi level *E*_F_ was calculated by the experimental Seebeck coefficient,^19^ and *m*_b_* was obtained from the ref. 31. At 300 K, *l*_c_ is as approximately three times large as the lattice parameter (≈5.94 Å) of FeNb_0.8_Ti_0.2_Sb materials due to the large effective mass, demonstrating that the charge carriers in this heavy‐band system are almost localized. Even though the sample BM‐16 h has the smallest grain size of ≈140 nm, it is still about two orders of magnitude higher than the calculated *l*_c_. As a result, the grain refinement only has negligible effect on the carrier mobility of FeNb_0.8_Ti_0.2_Sb (Figure 2b). By increasing Ti content to 25%, the *σ* of sample FeNb_0.75_Ti_0.25_Sb (*x* = 0) is slightly increased, compared with that of FeNb_0.8_Ti_0.2_Sb, resulting from the increased *p*_H_ (S1, Supporting Information). But the *μ*_H_ of FeNb_0.75_Ti_0.25_Sb has only a trivial change compared with that of FeNb_0.8_Ti_0.2_Sb, implying that the nanoscale precipitates may have negligible effect on the charge carrier transport.

**Figure 3:**
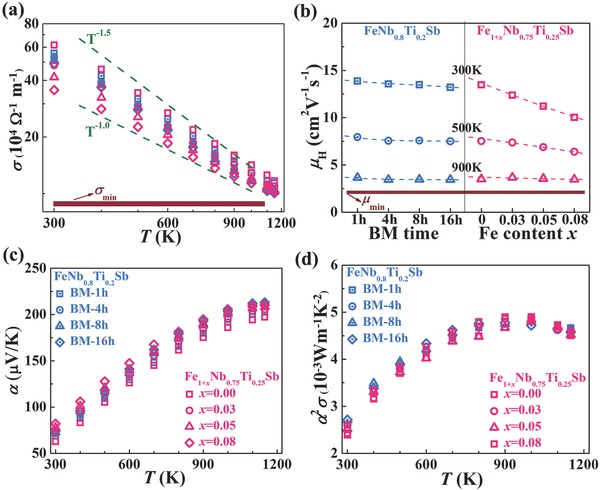
Temperature dependences of a) electrical conductivity *σ*, c) Seebeck coefficient *α*, and d) power factor *α* ^2^ *σ* for the BM FeNb_0.8_Ti_0.2_Sb and Fe_1+_ *_x_*Nb_0.75_Ti_0.25_Sb samples. b) Hall carrier mobility *μ*_H_ versus BM time and Fe content at different temperatures. The minimum electrical conductivity *σ*_min_ and carrier mobility *μ*_min_ in a) and b) were calculated based on the Ioffe–Regel criterion.^35^

However, for the Fe_1+_ *_x_*Nb_0.75_Ti_0.25_Sb samples with excess Fe, the *σ* has an obvious reduction with increasing Fe content, especially near room temperature (Figure 3a). For sample *x* = 0, the *σ* approximately follows a temperature dependence of *T* ^−1.5^ while the *σ* of sample *x* = 0.08 approaches the *T* ^−1.0^ dependence, indicating that Fe excess may enhance the alloying scattering of carriers. Hall measurement shows that both the decreased hole concentration and mobility (Figure S2 and S1, Supporting Information) lead to the decreased *σ*. Excess Fe may enter into the interstitial tetrahedral sites of the crystal structure to supply electrons, and make the hole concentration decrease. This phenomenon is similar to the scenario happened in another HH compound Ni_1+_ *_y_*ZrSn, in which excess Ni had been proven to enter into the interstitial tetrahedral sites and generate electrons.^28^, ^39^

At high temperatures the *σ* decrease with increasing Fe content for Fe_1+_ *_x_*Nb_0.75_Ti_0.25_Sb tends to be slower (Figure 3a), due to the slower decrease in high temperature *μ*_H_. As shown in Figure 3b, the room temperature *μ*_H_ of sample *x* = 0.08 has a ≈30% reduction compared with that of *x* = 0, but only ≈17% reduction at 500 K and almost unchanged at 900 K. By the Ioffe–Regel criterion that the lowest distance for metallic conduction is close to lattice constant, the minimum electrical conductivity *σ*_min_ and the minimum carrier mobility *μ*_min_ can be roughly estimated using the formulas^35^: *σ*_min_ = 0.33 *e* ^2^ *p* ^2/3^ *a*/*ℏ* and *μ*_min_ = *σ*_min_/*p*, where *e*, *p*, *a* and *ℏ* are the unit charge, carrier concentration, lattice parameter, and the reduced Planck constant, respectively. The calculated *σ*_min_ and *μ*_min_ of Fe_1+_ *_x_*Nb_0.75_Ti_0.25_Sb samples are displayed in Figure 3a,b, which suggests that at high temperatures this system is approaching the Ioffe–Regel limit and thus excess Fe will not notably enhance the carrier scattering and reduce the *μ*_H_. Figure 3c shows that the Seebeck coefficient *α* of the FeNb_0.8_Ti_0.2_Sb and Fe_1+_ *_x_*Nb_0.75_Ti_0.25_Sb samples display an increasing trend with temperature, which is typical behavior for degenerate semiconductors. The slight increase in *α* with increasing Fe content can be observed, corresponding to the decreased hole concentration (Figure S2, Supporting Information).

The above analysis shows that the grain refinement only has negligible effects on carrier transport of p‐type heavy‐band FeNbSb system due to the intrinsically low carrier mean free path. This phenomenon is rarely observed in traditional TE materials. Fe excess leads to a weak *μ*_H_ decrease at low temperatures, but almost has no effect at high temperatures. As a result, the introduced hierarchical phonon scattering centers have negligible effects on carrier transport of p‐type FeNbSb system and therefore the power factor *α* ^2^ *σ* is almost unchanged in the whole temperature range, as shown in Figure 3d.

The effect of hierarchical phonon scattering centers on thermal conductivity of p‐type FeNbSb is displayed in **Figure** **4**. The lattice thermal conductivity *κ*_L_ is obtained by subtracting the electronic component *κ*_e_ from the total *κ* and *κ*_e_ is calculated via *κ*_e_ = *LσT*, where *L* is the Lorenz number and can be calculated under the SPB approximation.^25^ As expected, the *κ* of the FeNb_0.8_Ti_0.2_Sb samples decreases with increasing BM time, ≈17% reduction at room temperature for sample BM‐16h, compared with that of sample BM‐1h (Figure 4a), which results from the both reduced *κ*_e_ (Figure 4b) and *κ*_L_ (Figure 4c). For FeNb_0.75_Ti_0.25_Sb (*x* = 0), although Ti‐rich nanoscale precipitates exist in the matrix, the *κ*_L_ of the sample has only a slight change compared with that of FeNb_0.8_Ti_0.2_Sb (BM‐8h), indicating that the introduced Ti‐rich nanoscale precipitates have a relatively weak effect on the *κ*_L_, which may be due to the relatively small contents of precipitates in the matrix.

**Figure 4:**
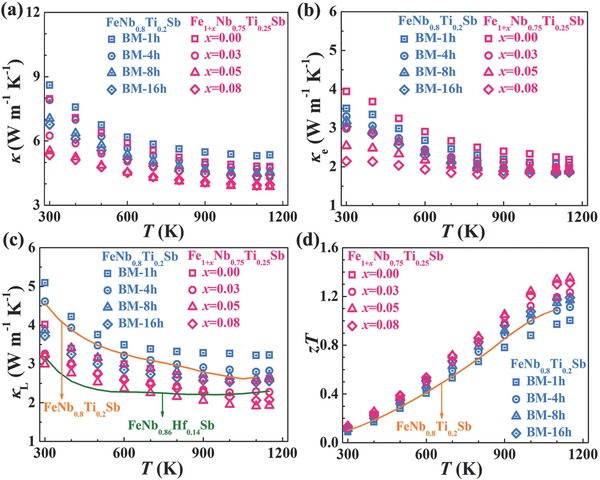
Temperature dependence of a) thermal conductivity *κ*, b) electronic thermal conductivity *κ*_e_, c) lattice thermal conductivity *κ*_L_ and d) *zT* values for the BMed FeNb_0.8_Ti_0.2_Sb and Fe_1+_ *_x_*Nb_0.75_Ti_0.25_Sb samples. The data lines in c) and d) show the *κ*_L_ and *zT* for FeNb_0.8_Ti_0.2_Sb and FeNb_0.86_Hf_0.14_Sb obtained in the refs. 31, 32.

An obvious decrease in *κ* is found for the Fe_1+_ *_x_*Nb_0.75_Ti_0.25_Sb samples with increasing Fe content (Figure 4a). For example, ≈30% reduction at room temperature is observed for *x* = 0.05, compared with *x* = 0. At low temperature, a large decrease in *κ*_e_ (Figure 4b) with increasing Fe content is the dominated reason for the large *κ* decrease. At high temperatures, the decrease in *κ*_e_ with increasing Fe content tends to be slower, consistent with the change of *σ* in Figure 3a. The *κ*_L_ obviously decreases with increasing Fe content as shown in Figure S3 (Supporting Information), mainly resulting from the additionally enhanced point defect scattering of phonons. The *κ*_L_ of *x* = 0.08 is ≈6% higher than that of *x* = 0.05, which may result from the uncertain measurement of thermal conductivity and estimation of Lorenz parameter, or Fe precipitation.

In short, the introduced hierarchical phonon scattering centers indeed significantly enhance the phonon scattering and decrease *κ*_L_ in p‐type FeNbSb. As a result, the *zT* of the samples is obviously enhanced. The maximum *zT* of ≈1.34 is obtained at 1150 K for the grain‐refined Fe_1.05_Nb_0.75_Ti_0.25_Sb (Figure 4d), ≈30% higher than that of FeNb_0.8_Ti_0.2_Sb with the shortest BM time. Considering that Ti is much cheaper and more abundant than Hf, the Ti‐doped Fe_1.05_Nb_0.75_Ti_0.25_Sb system should be great promising for large‐scale power generation application.

In summary, hierarchical phonon scattering is suggest to be effective in reducing the lattice thermal conductivity and enhancing the figure of merit of heavy‐band TE materials, and p‐type heavy‐band FeNbSb half‐Heusler system with intrinsically low carrier mean free path is demonstrated as a paradigm in this work. By combining the sub‐microscale grain boundaries, atomic‐scale point defects and electron–phonon interaction, hierarchical phonon scattering centers are concurrently introduced into the p‐type Ti‐doped FeNbSb, which have almost negligible effect on the carrier transport but contribute to a great reduction in the lattice thermal conductivity. Therefore, a high *zT* of 1.34 was obtained at 1150 K for the Fe_1.05_Nb_0.75_Ti_0.25_Sb compound with intentionally introduced hierarchical scattering centers. These results highlight the efficacy of hierarchical phonon scattering in improving the performance of heavy‐band TE system.

## Experimental Section

*Methods*: The ingots with nominal composition FeNb_0.8_Ti_0.2_Sb and Fe_1+_ *_x_*Nb_0.75_Ti_0.25_Sb (*x* = 0–0.08) were prepared by levitation melting of stoichiometric amount of Fe (piece, 99.97%), Nb (foil, 99.8%), Ti (rod, 99.99%) and Sb (block, 99.999%) under an argon atmosphere for 3 min and then remelted three times to ensure homogeneity. The ingots with nominal composition FeNb_0.8_Ti_0.2_Sb were subjected to a mechanical ball‐milling (BM) process (Mixer Mill MM200, Retsch) from 1 to 16 h under argon protection to obtain the powders with different sizes. The ingots with nominal composition Fe_1+_ *_x_*Nb_0.75_Ti_0.25_Sb were subjected to a BM process for 8 h. The obtained powders were loaded into the graphite die and compacted by spark plasma sintering (SPS‐1050, Sumitomo Coal Mining Co.) at 1123 K for 10 min under 65 MPa in vacuum. The as‐sintered samples, of which the relative densities were found to be ≈95%, were annealed at 1023 K for 2 d.

*Characterization*: Phase structures of the samples were investigated by X‐ray diffraction (XRD) on a RigakuD/MAX‐2550PC diffractometer using Cu Kα radiation (*λ*_0_ = 1.5406 Å). The diffraction peaks of all samples could be indexed to a single‐phase half‐Heusler structure and no obvious impurities exist in the samples. The chemical compositions (S1, Supporting Information) were checked by electron probe microanalysis (EPMA, JEOL, JXA‐8100). The XRD patterns of the BMed FeNb_0.8_Ti_0.2_Sb and Fe_1+_ *_x_*Nb_0.75_Ti_0.25_Sb samples show a single phase that can be indexed to the HH phase with a cubic MgAgAs‐type crystal structure (Figure S4, Supporting Information). The freshly fractured surfaces of the samples were observed by scanning electron microscopy (SEM) to show the particle size. Transmission electron microscopy (TEM, JEOL JEM‐3000F) was also performed to observe the possible nanostructures.

*Measurements*: The room temperature Hall coefficients were measured using a Mini Cryogen Free Measurement System (Cryogenic Limited, UK). The carrier concentration *p*_H_ was calculated by *p*_H_ = 1/*eR*_H_, where *e* is the unit charge and *R*_H_ is the Hall coefficient. The estimated error of Hall coefficient is within ±10%. The carriers mobility *μ*_H_ was calculated by *μ*_H_ = *σR*_H_. The Seebeck coefficient and electrical conductivity from 300—1150 K were measured on a commercial Linseis LSR‐3 system using a differential voltage/temperature technique and a DC four‐probe method. The accuracy is ±5% and ±3%, respectively. The thermal conductivity *κ* was calculated by using *κ* = *DρC*_p_, where *ρ* is the sample density estimated by the Archimedes method and. The thermal diffusivity *D* and specific heat *C*_p_ were measured by a laser flash method on Netzsch LFA457 instrument with a Pyroceram standard. The accuracy is ±3% and ±5%, respectively. The combined uncertainty for determining *zT* is less than 20%. The thermal stability of the samples was checked through the high‐temperature annealing treatment at 1023 K (Figure S5, Supporting Information). Only negligible change in TE properties was found with increased annealing time up to 10 d, indicating the good thermal stability for the samples with hierarchical phonon scattering centers.

## Supporting information

As a service to our authors and readers, this journal provides supporting information supplied by the authors. Such materials are peer reviewed and may be re‐organized for online delivery, but are not copy‐edited or typeset. Technical support issues arising from supporting information (other than missing files) should be addressed to the authors.

Supplementary
