# Supplementary material for: Enhancing the Figure of Merit of Heavy‐Band Thermoelectric Materials Through Hierarchical Phonon Scattering
Source: Adv Sci (Weinh). 2016 Mar 15;3(8):1600035. doi: 10.1002/advs.201600035 (PMC5069587; doi:10.1002/advs.201600035)
Supplement: Supplementary file 1 — Supplementary [file ADVS-3-0m-s001.pdf]

Copyright WILEY-VCH Verlag GmbH & Co. KGaA, 69469 Weinheim, Germany, 2016.

ADVANCED SCIENCE
Open Access

Supporting Information

for *Adv. Sci.*, DOI: 10.1002/advs.201600035

Enhancing the Figure of Merit of Heavy-Band Thermoelectric Materials Through Hierarchical Phonon Scattering

*Chenguang Fu, Haijun Wu, Yintu Liu, Jiaqing He, Xinbing Zhao, and Tiejun Zhu**

WILEY-VCH

Copyright WILEY-VCH Verlag GmbH & Co. KGaA, 69469 Weinheim, Germany, 2013.

Supporting Information

Enhancing the Figure of Merit of Heavy-Band Thermoelectric Materials through Hierarchical Phonon Scattering

*Chenguang Fu, Haijun Wu, Yintu Liu, Jiaqing He, Xinbing Zhao and Tiejun Zhu**

C. G. Fu, Y. T. Liu, Prof. X. B. Zhao, Prof. T. J. Zhu
State Key Laboratory of Silicon Materials, Department of Materials Science and Engineering, Zhejiang University, Hangzhou 310027, China
*E-mail: zhutj@zju.edu.cn

H. J. Wu, Prof. J. Q. He
Department of Physics, South University of Science and Technology of China, Shenzhen 518055, China

H. J. Wu
Department of Materials Science and Engineering, National University of Singapore, 7 Engineering Drive 1, Singapore 117575, Singapore

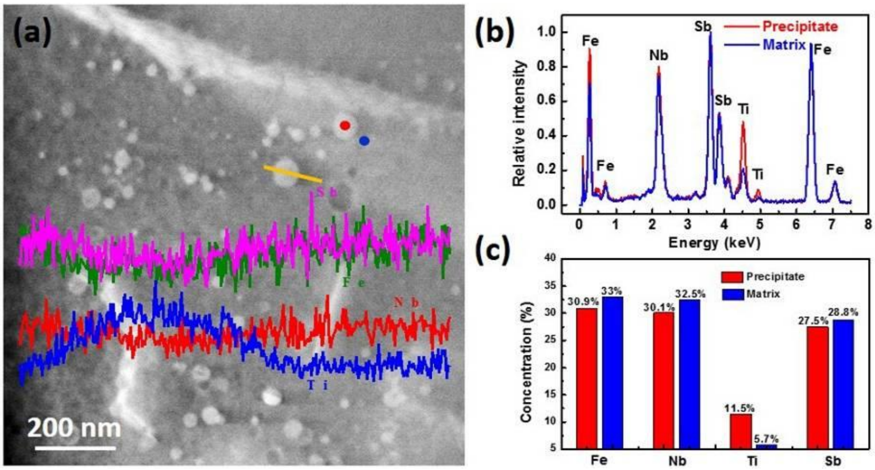

Figure S1. TEM analysis for sample $Fe_{1.05}Nb_{0.75}Ti_{0.25}Sb$. (a) Medium-magnification HAADF image and inset EDS line spectra; (b) Point EDS spectra for precipitates (red) and matrix (blue); (c) The compositions of precipitates and matrix, averaged from several point EDS results.

Table S1. The EPMA composition and room temperature electrical conductivity σ, Seebeck coefficient α, Hall carrier concentration p_H, Hall carrier mobility μ_H, and the calculated carrier mean free path l_c for the BM $FeNb_{0.8}Ti_{0.2}Sb$ and $Fe_{1+x}Nb_{0.75}Ti_{0.25}Sb$ samples.

Samples	EPMA composition	σ ($10^4\Omega^{-1}m^{-1}$)	α (μV/K)	p_H ($10^{20}cm^{-3}$)	μ_H ($cm^2V^{-1}s^{-1}$)	l_c (Å) 300K
BM-1h	$Fe_{1.005}Nb_{0.801}Ti_{0.195}Sb_{0.999}$	60.6	68.5	27.3	13.9	19.5
BM-4h	$Fe_{1.010}Nb_{0.796}Ti_{0.194}Sb$	57.5	71.5	26.5	13.6	18.7
BM-8h	$Fe_{1.014}Nb_{0.798}Ti_{0.194}Sb_{0.994}$	55.6	72.8	25.8	13.5	18.4
BM-16h	$Fe_{1.015}Nb_{0.800}Ti_{0.191}Sb_{0.994}$	53.5	74.3	25.3	13.2	17.8
x=0	$Fe_{1.004}Nb_{0.769}Ti_{0.228}Sb_{0.999}$	61.6	62.8	28.5	13.5	19.8
x=0.03	$Fe_{1.029}Nb_{0.778}Ti_{0.226}Sb_{0.998}$	48.2	73.6	24.3	12.4	16.8
x=0.05	$Fe_{1.048}Nb_{0.772}Ti_{0.230}Sb$	41.6	77.8	23.2	11.2	14.8
x=0.08	$Fe_{1.045}Nb_{0.777}Ti_{0.232}Sb_{0.996}$	35.6	82.0	22.2	10.0	12.9

Discussion on the solubility of Ti

The solubility of Ti in FeNbSb is verified by combining the two aspects: 1) from the change in the lattice parameter of $FeNb_{1-x}Ti_xSb$ sample (as shown in the Supporting Information of Ref. 1(Fig. S9)),[1] the lattice parameter of $FeNb_{1-x}Ti_xSb$ follow the Vegard's law up to x=0.2 while the lattice parameter of x=0.24 deviates from the Vegard's law, indicating the solubility of Ti should lie in the range of 20% - 24%; 2) furthermore, the EPMA results in the Table S1 show that all the $Fe_{1+x}Nb_{0.75}Ti_{0.25}Sb$ samples display similar actual Ti content of ~23%. Therefore, collaborating the above two points, the solubility of Ti in FeNbSb is estimated to be ~23%.

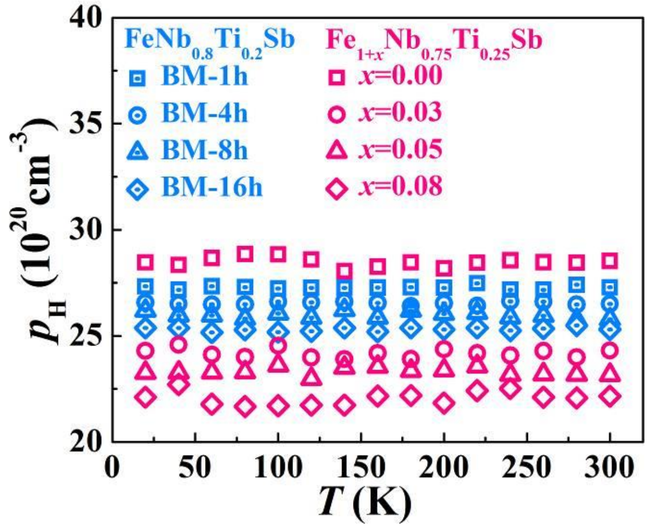

Figure S2. Temperature dependence of Hall carrier concentration for the BMed $FeNb_{0.8}Ti_{0.2}Sb$ and $Fe_{1+x}Nb_{0.75}Ti_{0.25}Sb$ samples.

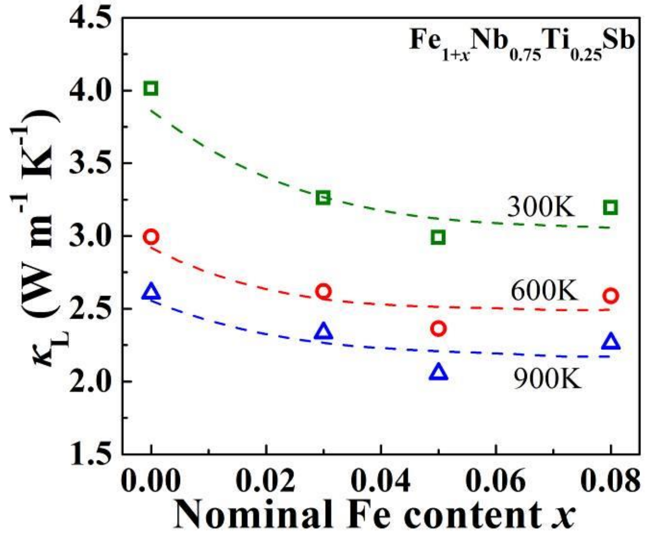

Figure S3. Nominal Fe content dependence of lattice thermal conductivity for $Fe_{1+x}Nb_{0.75}Ti_{0.25}Sb$ samples at 300K, 600K and 900K, respectively. The dash lines shows the decreasing trend of κ_L.

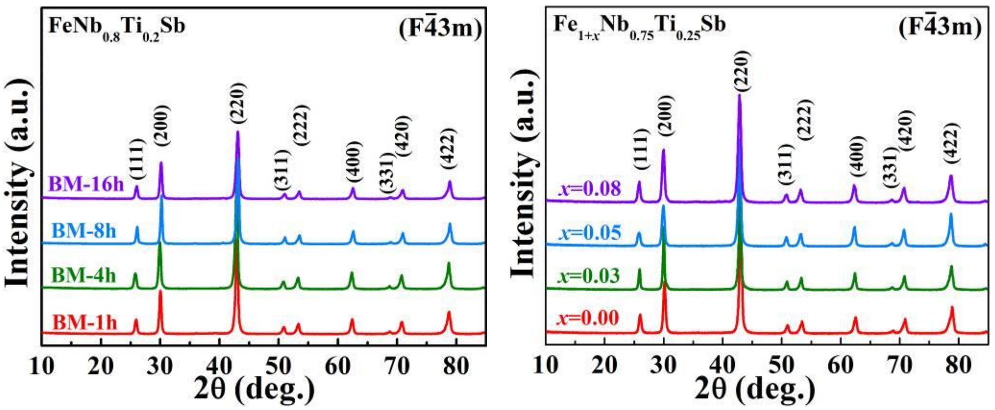

Figure S4. XRD patterns of the BMed $FeNb_{0.8}Ti_{0.2}Sb$ and $Fe_{1+x}Nb_{0.75}Ti_{0.25}Sb$ samples. All XRD patterns can be indexed to the cubic MgAgAs-type half-Heusler crystal structure.

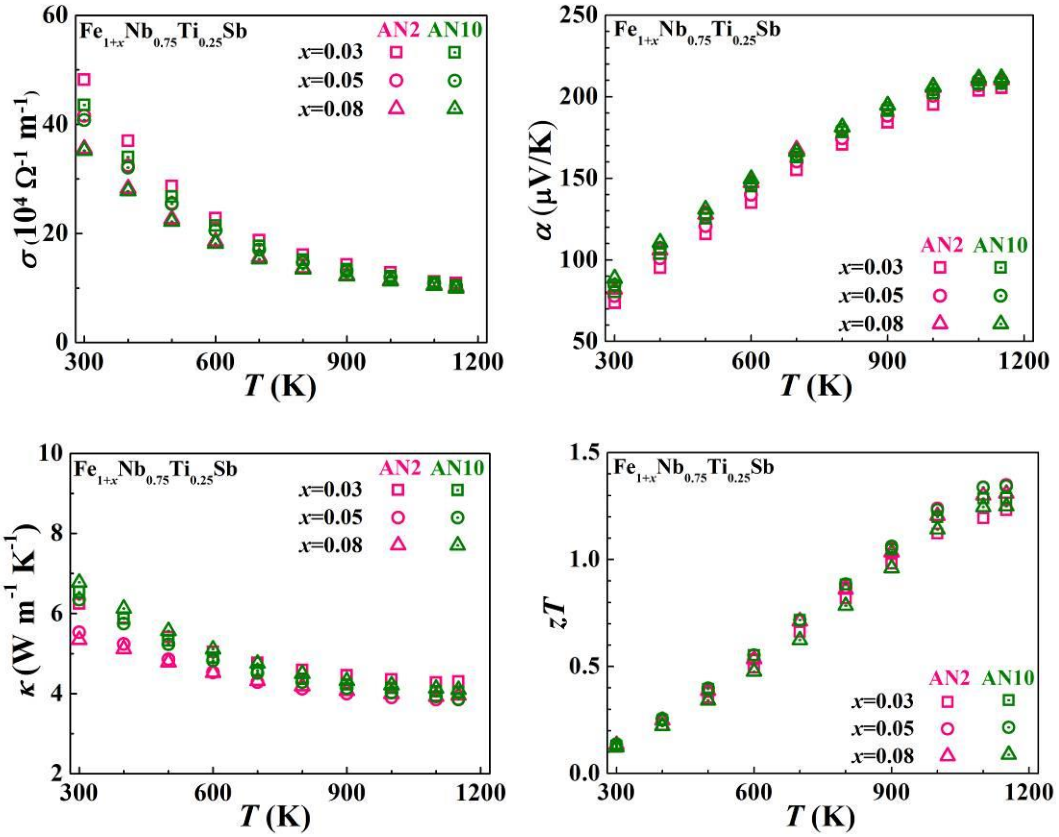

Figure S5. TE properties versus temperature for $Fe_{1+x}Nb_{0.75}Ti_{0.25}Sb$ samples annealed (AN) for 2 and 10 days under 1023K, respectively. (a) Electrical conductivity, (b) Seebeck coefficient, (c) Thermal conductivity, and (d) *zT* value.

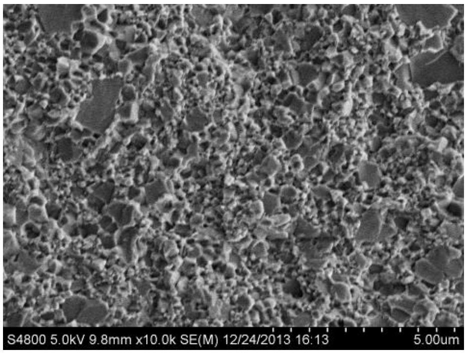

Figure S6. SEM image of the fractured surface for $Fe_{1.05}Nb_{0.75}Ti_{0.25}Sb$ sample milled for 8h.

Spectral lattice thermal conductivity calculation

The spectral lattice thermal conductivity displayed in Figure 1b is calculated based on the following process:

In 1959, J. Callaway had proposed a phenomenological model about the lattice thermal conductivity, which can be expressed as:[2]

$$\kappa_L = \frac{k_B}{2\pi^2 \upsilon}(\frac{k_B T}{\hbar})^3 \int_0^{\theta_D/T} \frac{x^4 e^x}{\tau^{-1}(e^x - 1)^2} dx \qquad (1)$$

where $x = \hbar\omega / k_B T$ is the reduced phonon frequency, k_B the Boltzmann constant, $\hbar$ the reduced Planck constant, θ_D the Debye temperature, υ the sound velocity, and τ the effective relaxation time. In this work, τ is expressed as

$$\tau^{-1} = A_1\omega^4 + B\omega^2 T \exp(-\theta_D / 3T) + C\omega^2 + \frac{\upsilon}{d} + A_2\omega^4 \qquad (2)$$

where d is the grain size and υ/d represents boundary scattering, A_1 and A_2 are the prefactors for point defect (PD) scattering relaxation time due to Ti doping and Fe excess, respectively, and B is the prefactor for phonon-phonon Umklapp (U) scattering relaxation time, and C is the prefactor for electron-phonon (EP) scattering

relaxation time.

For the polycrystalline pure FeNbSb, the dominated phonon scattering mechanism should be the phonon-phonon U scattering and boundary scattering. Therefore, through fitting the κ_L of polycrystalline FeNbSb, we can obtain the prefactor B of U scattering relaxation time. Scattering by point defects arises from both mass and strain differences within the lattice. In the simple case of alloying, $\tau_{PD}^{-1} = \frac{V\omega^4}{4\pi\upsilon^3}(\Gamma_M + \Gamma_S)$, where Γ_M and Γ_S are the disorder scattering parameters due to mass and strain field fluctuations, respectively. The detailed calculation of point defects scattering can be found elsewhere.[3,4] For $FeNb_{0.8}Ti_{0.2}Sb$, the existing phonon scattering sources should contain the U process, boundary, point defects and electron-phonon interaction. The boundary scattering and point defects scattering relaxation times can be calculated independently, while U scattering relaxation time can be obtained from fitting the polycrystalline FeNbSb. Therefore, through fitting the κ_L of $FeNb_{0.8}Ti_{0.2}Sb$, the prefactor C of EP scattering relaxation time can be obtained. For $Fe_{1.05}Nb_{0.75}Ti_{0.25}Sb$, the same C is used since it has similar hole concentration with $FeNb_{0.8}Ti_{0.2}Sb$. Thus, the prefactor A_2 for point defect scattering relaxation time due to Fe excess can be obtained by fitting the κ_L of $Fe_{1.05}Nb_{0.75}Ti_{0.25}Sb$.

To study the effect of different phonon scattering mechanism on the lattice thermal conductivity, the spectral lattice thermal conductivity κ_s is expressed as[5,6]

$$\kappa_s = \frac{k_B}{2\pi^2\upsilon}\left(\frac{k_B T}{\hbar}\right)^3 \frac{x^4 e^x}{\tau^{-1}(e^x - 1)^2} \qquad (3)$$

Thus, by introducing different phonon scattering relaxation time as shown in Table S2 into equation (3), the corresponding spectral lattice thermal conductivity can be obtained.

Table S2. The important parameters used for estimating the lattice thermal conductivity of $Fe_{1.05}Nb_{0.75}Ti_{0.25}Sb$.

Scattering type	***parameter***	***value***
Umklapp scattering	B (10^{-18} s/K)	2.6
Point defect scattering due to Ti doping	The strain field adjust parameter ε	90
	A_1 (10^{-43} s^3)	7.6
	Sound velocity υ_s (m/s)	3433
	Debye temperature θ_D (K)	394
Point defect scattering due to Fe excess	A_2 (10^{-43} s^3)	8.8
Electron-phonon interaction	C (10^{-16} s)	13
Boundary scattering	Average grain size d (nm)	190

References

[1] C. G. Fu, T. J. Zhu. Y. T. Liu. H. H. Xie, X. B. Zhao, *Energy Environ. Sci.* **2015**, *8*, 216.

[2] J. Callaway, *Phys. Rev.* **1959**, *113*, 1046.

[3] C. G. Fu, H. H. Xie, T. J. Zhu, J. Xie, and X. B. Zhao, *J. Appl. Phys.* **2012**, *112*, 124915.

[4] J. Yang, G. P. Meisner, L. Chen, *Appl. Phys. Lett.* **2004**, *85*, 1140.

[5] E. S. Toberer, A. Zevalkink, G. J. Snyder, *J. Mater. Chem.* **2011**, *21*, 15843.

[6] T. J. Zhu, C. G. Fu, H. H. Xie, Y. T. Liu, B. Feng, J. Xie, X. B. Zhao, *EPL* **2013**, *104*, 46003.
